# Supplementary material for: Neurogenomic insights into paternal care and its relation to territorial aggression
Source: Nat Commun. 2019 Sep 30;10:4437. doi: 10.1038/s41467-019-12212-7 (PMC6768867; doi:10.1038/s41467-019-12212-7)
Supplement: Supplementary file 1 — Supplementary Information [file 41467_2019_12212_MOESM1_ESM.pdf]

Supplementary Information

for

# Neurogenomic insights into paternal care and its relation to territorial aggression

Bell et al

(a)

| Gene | FDR in set 1 | FDR in set 2 | Unique to? | Shared? |
|---|---|---|---|---|
| 1 | 0.00001 | 0.001 | | Y |
| 2 | 0.0001 | 0.001 | | Y |
| 3 | 0.03 | 0.002 | 2 | Y |
| 4 | 0.00003 | 0.3 | 1 | N |
| 5 | 0.00004 | 0.03 | | N |
| 6 | 0.005 | 0.04 | | N |
| 7 | 0.006 | 0.05 | | N |
| 8 | 0.007 | 0.06 | | N |
| 9 | 0.05 | 0.00001 | | N |
| 10 | 0.001 | 0.02 | 1 | N |
| 11 | 0.02 | 0.5 | | N |
| 12 | 0.03 | 0.03 | | N |
| 13 | 0.04 | 0.3 | | N |
| 14 | 0.05 | 0.05 | | N |
| 15 | 0.06 | 0.06 | | N |
| 16 | 0.25 | 0.0001 | 2 | N |
| 17 | 0.08 | 0.4 | | N |
| 18 | 0.1 | 0.3 | | N |
| 19 | 0.3 | 0.2 | | N |
| 20 | 0.4 | 0.1 | | N |

(b)

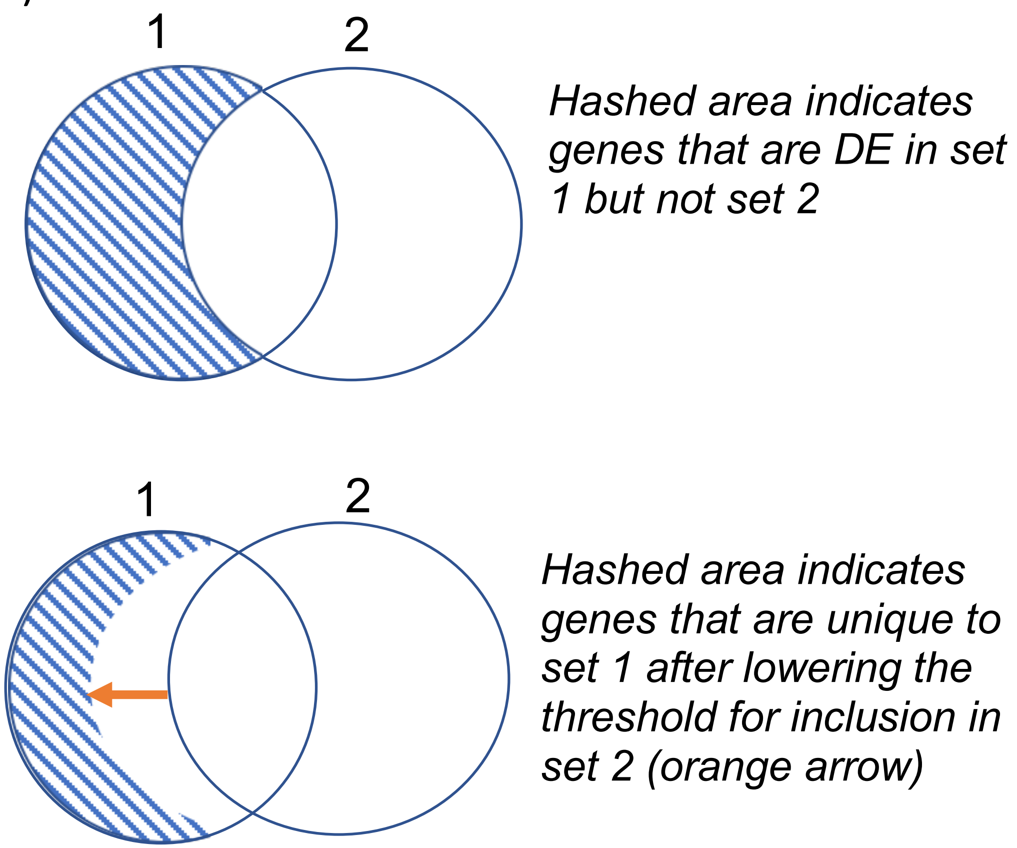


**Supplementary Figure 1.**

**Supplementary Figure 1.** Procedure for identifying genes that are unique to a particular set of genes (i.e. nonshared). a) The table shows hypothetical FDR values of the differential expression between the control and experimental condition in two different comparisons for 20 different genes. In order for a gene to be considered unique to a particular set, it had to be DE in that set at FDR<0.01 and it had to be clearly not differentially expressed in another set, i.e. at FDR>0.2. In contrast, genes that were shared between sets were DE at FDR<0.01 in both sets. b) Venn diagrams to illustrate how increasing the FDR threshold in a non-focal set reduces the size of the gene set that is unique to the focal set. These hypothetical venn diagrams show the number of DEGs in two different sets (1 and 2) and the overlap between them. In the top panel, the hashed area indicates genes that are DE in set 1 at FDR<0.01 but are not DE in set 2 at FDR<0.01. To guard against the possibility that some of the genes in the hashed area just barely passed the threshold for differential expression in set 2, we expanded the gene set in set 2 to include genes that were DE at FDR<0.2, as indicated by the orange arrow. The hashed area now indicates genes that we consider to be unique to set 1; note that the size of the hashed area in the bottom panel is considerably smaller than the hashed area in the top panel, because the unique genes had to pass a more stringent filter for inclusion.

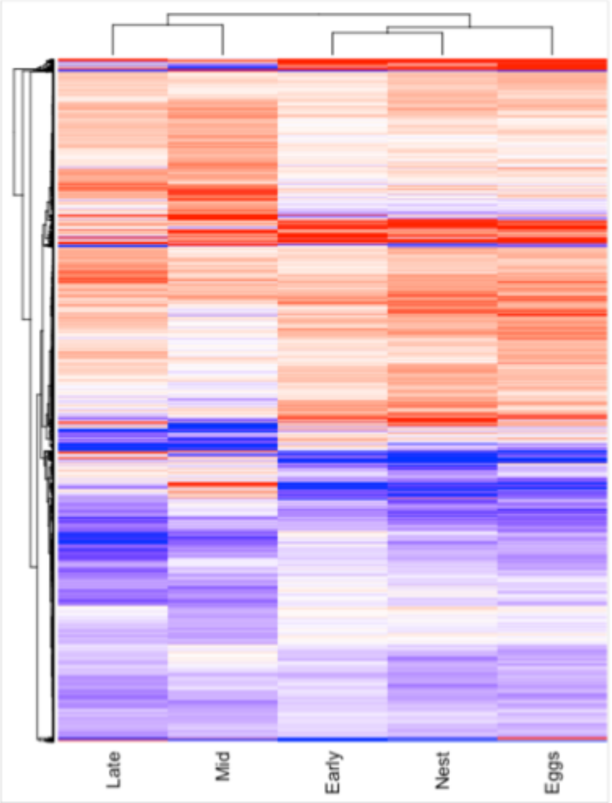


**Supplementary Figure 2.**

**Supplementary Figure 2.** Hierarchical clustering of logFC of all of the differentially expressed genes between the control and experimental conditions during the in the nest, eggs, early, mid and late stages (1674 genes).

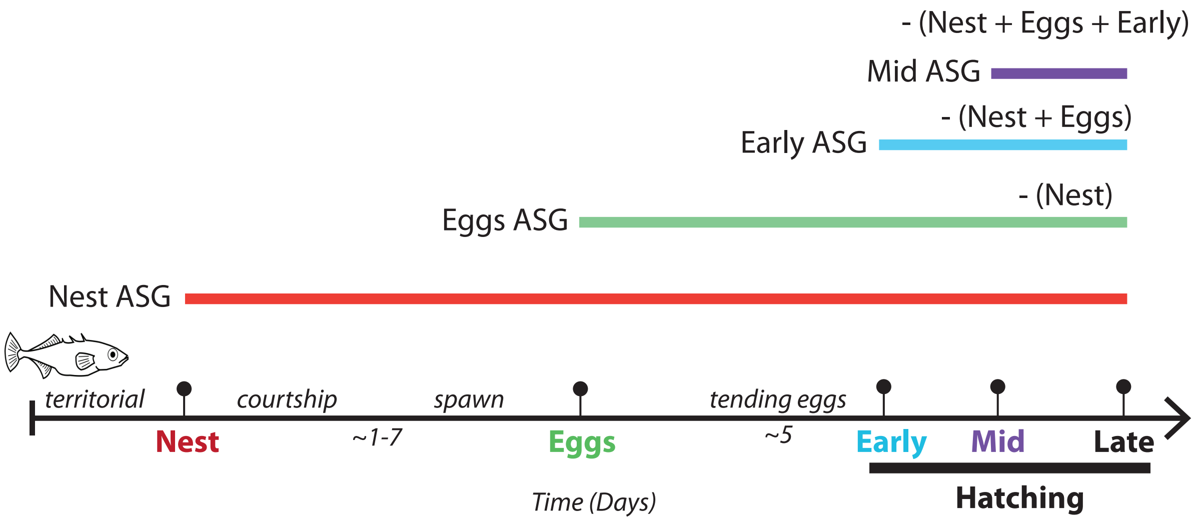


**Supplementary Figure 3.**

**Supplementary Figure 3. Conceptual overview of added shared genes (ASG)**. The general timeline for the experiment is shown at the bottom of the figure. In order to identify genes which were added to a stage and which remained differentially expressed between the control and experimental group in subsequent stages (added shared genes), we identified shared DEGs between a stage of interest and at least one subsequent stage, and then removed from this list any genes that were differentially expressed during a preceding stage. For example, "mid added shared genes" include genes that were differentially expressed during the middle and late stages, but were not differentially expressed in any of the preceding stages. Stickleback drawing by MB.

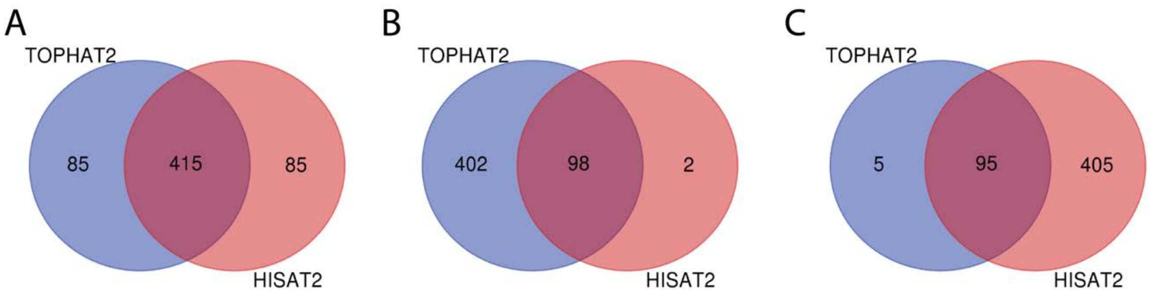


**Supplementary Figure 4**

**Supplementary Figure 4. Comparing the results of differential gene expression analysis using TopHat2 versus HISAT2**. Shown are representative results for differential expression in diencephalon between males during the nesting stage versus the control group. (A) Shows the overlap between the top 500 differentially expressed genes according to TopHat2 (blue) and HISAT2 (red). The two tools generated very similar results, with 83% of the top 500 DEGs shared between the two tools. The Venn diagrams on the right show how many of the top 100 ranked genes according to one tool were within the top 500 ranked genes according to the other tool. (B) 98 of the top 100 genes using HISAT2 are present in the top 500 genes using TopHat2. (C) 95 of the top 100 genes using Tophat2 are present in the top 500 genes using HiSat2. The large overlap suggests that the genes that are ranked highly by one tool (i.e. in the top 100) are also ranked highly by the other tool (i.e. in the top 500); the ranks are not exactly the same between the two tools because of different alignment protocols.
