# Supplementary material for: Neurogenomic insights into paternal care and its relation to territorial aggression
Source: Nat Commun. 2019 Sep 30;10:4437. doi: 10.1038/s41467-019-12212-7 (PMC6768867; doi:10.1038/s41467-019-12212-7)
Supplement: Supplementary file 3 — Reporting Summary [file 41467_2019_12212_MOESM3_ESM.pdf]

nature research

Corresponding author(s): Alison Bell

Last updated by author(s): Jul 22, 2019

Reporting Summary

Nature Research wishes to improve the reproducibility of the work that we publish. This form provides structure for consistency and transparency in reporting. For further information on Nature Research policies, see Authors & Referees and the Editorial Policy Checklist.

Statistics

For all statistical analyses, confirm that the following items are present in the figure legend, table legend, main text, or Methods section.

n/a	Confirmed	
☐	☒	The exact sample size (*n*) for each experimental group/condition, given as a discrete number and unit of measurement
☐	☒	A statement on whether measurements were taken from distinct samples or whether the same sample was measured repeatedly
☐	☒	The statistical test(s) used AND whether they are one- or two-sided<br>*Only common tests should be described solely by name; describe more complex techniques in the Methods section.*
☐	☒	A description of all covariates tested
☐	☒	A description of any assumptions or corrections, such as tests of normality and adjustment for multiple comparisons
☐	☒	A full description of the statistical parameters including central tendency (e.g. means) or other basic estimates (e.g. regression coefficient) AND variation (e.g. standard deviation) or associated estimates of uncertainty (e.g. confidence intervals)
☐	☒	For null hypothesis testing, the test statistic (e.g. *F*, *t*, *r*) with confidence intervals, effect sizes, degrees of freedom and *P* value noted<br>*Give P values as exact values whenever suitable.*
☒	☐	For Bayesian analysis, information on the choice of priors and Markov chain Monte Carlo settings
☐	☒	For hierarchical and complex designs, identification of the appropriate level for tests and full reporting of outcomes
☒	☐	Estimates of effect sizes (e.g. Cohen's *d*, Pearson's *r*), indicating how they were calculated

Our web collection on statistics for biologists contains articles on many of the points above.

Software and code

Policy information about availability of computer code

Data collection	No software was used to collect the data.
Data analysis	Codes are available on GitHub [https://github.com/bukhariabbas/stickleback-paternal-care].

For manuscripts utilizing custom algorithms or software that are central to the research but not yet described in published literature, software must be made available to editors/reviewers. We strongly encourage code deposition in a community repository (e.g. GitHub). See the Nature Research guidelines for submitting code & software for further information.

Data

Policy information about availability of data

All manuscripts must include a data availability statement. This statement should provide the following information, where applicable:

- Accession codes, unique identifiers, or web links for publicly available datasets
- A list of figures that have associated raw data
- A description of any restrictions on data availability

The datasets generated during and/or analysed during the current study are available in GEO (accession code number GSE134508 [https://www.ncbi.nlm.nih.gov/geo/query/acc.cgi?acc=GSE134508; Enter token wtwdkuqobnabpij into the box]. Codes are available on GitHub [https://github.com/bukhariabbas/stickleback-paternal-care]. All other relevant data is available upon request.

Field-specific reporting

Please select the one below that is the best fit for your research. If you are not sure, read the appropriate sections before making your selection.

☐ Life sciences ☐ Behavioural & social sciences ☒ Ecological, evolutionary & environmental sciences

For a reference copy of the document with all sections, see nature.com/documents/nr-reporting-summary-flat.pdf

Ecological, evolutionary & environmental sciences study design

All studies must disclose on these points even when the disclosure is negative.

Study description	The neurogenomic changes associated with paternal care were compared between males at different stages of paternal care versus control males. Neurogenomic profiling was carried out in two brain regions: diencephalon and telencephalon
Research sample	Wild-caught adult male sticklebacks
Sampling strategy	Sample sizes were based on comparable studies of neurogenomic changes associated with behavior, with n=5 individuals per condition
Data collection	Brain tissue was dissected, RNA extracted, libraries prepped and sequenced at the Keck Center at the University of Illinois.
Timing and spatial scale	All brain tissue samples were collected in summer 2013
Data exclusions	One sample was excluded because of low quality RNA and was not sequenced.
Reproducibility	The experimental samples were replicated with n=5 individuals per condition.
Randomization	Males were randomly assigned to either the control or experimental group
Blinding	Samples were analyzed blind

Did the study involve field work? ☐ Yes ☒ No

Reporting for specific materials, systems and methods

We require information from authors about some types of materials, experimental systems and methods used in many studies. Here, indicate whether each material, system or method listed is relevant to your study. If you are not sure if a list item applies to your research, read the appropriate section before selecting a response.

Materials & experimental systems

n/a	Involved in the study
☒	☐ Antibodies
☒	☐ Eukaryotic cell lines
☒	☐ Palaeontology
☐	☒ Animals and other organisms
☒	☐ Human research participants
☒	☐ Clinical data

Methods

n/a	Involved in the study
☒	☐ ChIP-seq
☒	☐ Flow cytometry
☒	☐ MRI-based neuroimaging

Animals and other organisms

Policy information about studies involving animals; ARRIVE guidelines recommended for reporting animal research

Laboratory animals	*For laboratory animals, report species, strain, sex and age OR state that the study did not involve laboratory animals.*
Wild animals	Adult wild animals were collected with minnow traps, transported via air overnight to the lab at the University of Illinois. Males were sacrificed for brain collection
Field-collected samples	Animals were collected in the field and studied in the lab under an IACUC-approved protocol
Ethics oversight	This study was approved by the University of Illinois IACUC under protocol #15077

Note that full information on the approval of the study protocol must also be provided in the manuscript.
