# Supplementary material for: Neurogenomic insights into paternal care and its relation to territorial aggression
Source: Nat Commun. 2019 Sep 30;10:4437. doi: 10.1038/s41467-019-12212-7 (PMC6768867; doi:10.1038/s41467-019-12212-7)
Supplement: Supplementary file 4 — Description of Additional Supplementary Files [file 41467_2019_12212_MOESM4_ESM.pdf]

Description of Additional Supplementary Files

Supplementary Data 1. Stickleback paternal care and territorial aggression DEGs. Each sheet reports the list of DEGs for a pairwise treatment vs control comparison in each condition.

Supplementary Data 2. Functional enrichment of paternal care DEGs with conditions in different columns; statistically significant p-values are shown.

Supplementary Data 3. Pairwise gene set intersections between different stages of paternal care along with their p-values.

Supplementary Data 4. Statistical significance of added shared genes (ASG) as assessed by ROAST. Results for the different brain regions of both stickleback and mouse are shown in different sheets. Each sheet contains a table which consists of seven columns. “Name” indicates the set being tested, “contrast” indicates the comparison against the set which will be tested. “NGenes” is number of genes in the set, “PropUp” is proportion of genes in set with $z > sqrt(2)$, “PropDown” is proportion of genes in set with $z < -sqrt(2)$, “Direction” is direction of change such as up or down, and “FDR” is a fdr of direction based test. A significant “FDR” suggests significantly differential expression of genes in the given set along with consistent direction.

Supplementary Data 5. Mouse maternal care DEGs at $FDR < 0.01$, each sheet reports the list of DEGs for a pairwise treatment vs control comparison in each condition.

Supplementary Data 6. Shows significant orthogroups along with their representative DEGs from stickleback and mouse. The sheet “summary” represents significance of overlaps across analogous brain regions as assessed by permutation tests. The sheet “Orthogroup_D_Hypothalamus” represents added shared gene orthogroups between diencephalon and hypothalamus. The sheet “Orthogroup_D_Hypothalamus” represents added shared gene orthogroups between telencephalon and hippocampus.

Supplementary Data 7. Functional enrichment of genes that were unique to either the paternal care or territorial aggression experiment.

Supplementary Data 8. Results of ASTRIX analysis to build a transcriptional regulatory network for the paternal care and territorial aggression DEGs. Shown are transcription factors and their targets that were enriched in both datasets.

Supplementary Data 9. Shows each library read count information along with the lane in which they were sequenced.
